# Induction of differentiation of the acute myeloid leukemia cell line (HL-60) by a securinine dimer

**DOI:** 10.1038/s41420-020-00354-3

**Published:** 2020-11-12

**Authors:** Wen Hou, Zhen-Ya Wang, Jing Lin, Wei-Min Chen

**Affiliations:** International Cooperative Laboratory of Traditional Chinese Medicine Modernization and Innovative Drug Development of Chinese Ministry of Education (MOE), College of Pharmacy, Jinan University, Guangzhou, 510632 PR China

**Keywords:** Acute myeloid leukaemia, Drug development

## Abstract

Differentiation therapy has been successfully applied clinically in cases of acute promyelocytic leukemia (APL), but few differentiation-induction agents other than all-trans retinoic acid (**ATRA**) have been discovered clinically. Based on our previously reported neuritogenic differentiation activity of synthetic dimeric derivatives of securinine, we explored the leukemia differentiation-induction activity of such as compound, **SN3-L6**. It was found that **SN3-L6** induces transdifferentiation of both acute myeloid leukemia (AML) and chronic myelogenous leukemia (CML) cells but unexpectedly, a new transdifferentiation pathway from APL cells to morphologically and immunologically normal megakaryocytes and platelets were discovered. **SN3-L6** fails to induce transdifferentiation of **ATRA**–produced mature granulocytes into megakaryocytes, indicating its selectivity between mature and immature cells. **SN3-L6** induces CML K562 cells to transdifferentiate into apoptotic megakaryocytes but without platelet formation, indicating a desirable selectivity between different leukemia cells. Our data illuminate a differentiation gap between AML cells and platelets, and promises applications in leukemia differentiation therapy strategy.

## Introduction

**Fig. 1:**
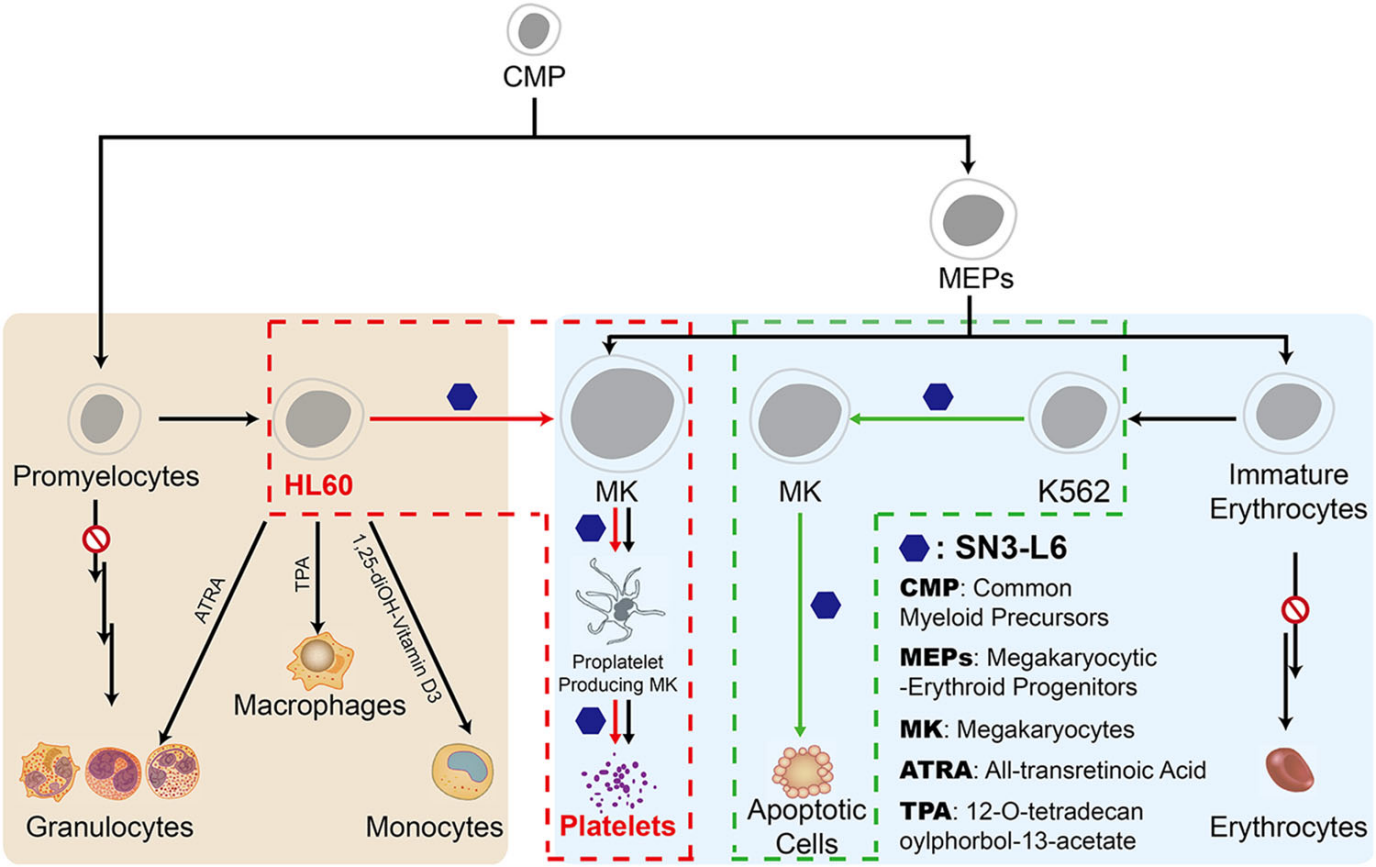
Normal and new differentiation pathways of hemocytes or induced differentiation pattern of leukemia cells by inductive agents. **SN3-L6** is a synthetic securinine derivative. Pathway indicated by black arrows is occurred in our marrow and reported by others. Pathway indicated by red arrows in rectangle with red dotted lines is newly discovered in this study, that HL60 cells were found for the first time to be induced by small molecule to form MK cells, and MK cells were successfully induced by small molecule to differentiate into platelets instead of apoptosis. Pathway indicated by green arrows in rectangle with green dotted lines is the differentiation pathway observed after **SN3-L6** induction that consistent with the induced differentiation reported by others.

Leukemia is characterized by a blockage of cell differentiation (Fig. 1). The most common method for treatment of leukemia involves chemotherapeutic agents that kill cancer cells but have a number of severely toxic side-effects. An alternative treatment of leukemia, especially acute promyelocytic leukemia (APL), a type of acute myeloid leukemia (AML), involves medicines that alter tumor growth by inducing terminal differentiation. Agents that act as terminal differentiation inducers include all-trans retinoic acid (**ATRA**) and arsenic trioxide (As_2_O_3_), that can cure APL^1–5^. With a low (<10%) 5-year survival rate, AML has the second-highest morbidity of all leukemia types^6^. Since **ATRA** was successfully applied clinically^7^, differentiation therapy became an effective treatment for APL in 2009, but few differentiation-induction agents other than **ATRA** have since been discovered clinically. It is well known that inducing APL cells to differentiate into mature granulocytes is the main pharmacologic action of **ATRA**. It has been reported that monocytic differentiation of HL60 cells can be induced by 1,25-dihydroxy vitamin D_3_^8,9^ and differentiation towards the macrophage lineage can be assisted by 12-*O*-tetradecanoylphorbol 13-acetate^10^ as indicated in Fig. 1. However, no other novel leukemia differentiation pathways have been reported to provide possible differentiation therapies for leukemia patients. The discovery of new differentiation pathways is therefore significant for the promotion of the discovery of novel differentiation inducers which could benefit different types of leukemia patients.

Securinine is an alkaloid found in the leaves of *Securinega*, *Phyllanthus*, and *Flueggea genera*^11^. Our previous research revealed the effective neuritogenic differentiation activity of some synthetic dimeric securinine derivatives^12,13^. Accordingly, we have explored the leukemia differentiation induction activity of synthetic securinine derivatives and found that a synthetic securinine derivative, **SN3-L6**, unexpectedly exhibited induction of transdifferentiation of AML cells. It induces HL60 cells to transdifferentiate into living megakaryocytes and to release platelets as a rectangle with red dotted lines indicated in Fig. 1. This is a novel and previously unreported transdifferentiation pathway for HL60 cells. **SN3-L6** can also induce K562 cells present in chronic myelogenous leukemia (CML), transdifferentiate them into megakaryocytes without the formation of platelets and is shown as a rectangle with green dotted lines indicated in Fig. 1. This is a pathway that has been reported elsewhere^14–16^. This newly discovered transdifferentiation pathway provides a tool with which to study the linkage of cells of origin between HL60 cells and megakaryocytes/platelets. The potential application value merits further study and it should be possible to develop new strategies for the treatment of leukemia based on this novel transdifferentiation pathway.

## Results

### SN3-L6 induced megakaryocyte formation

**Fig. 2:**
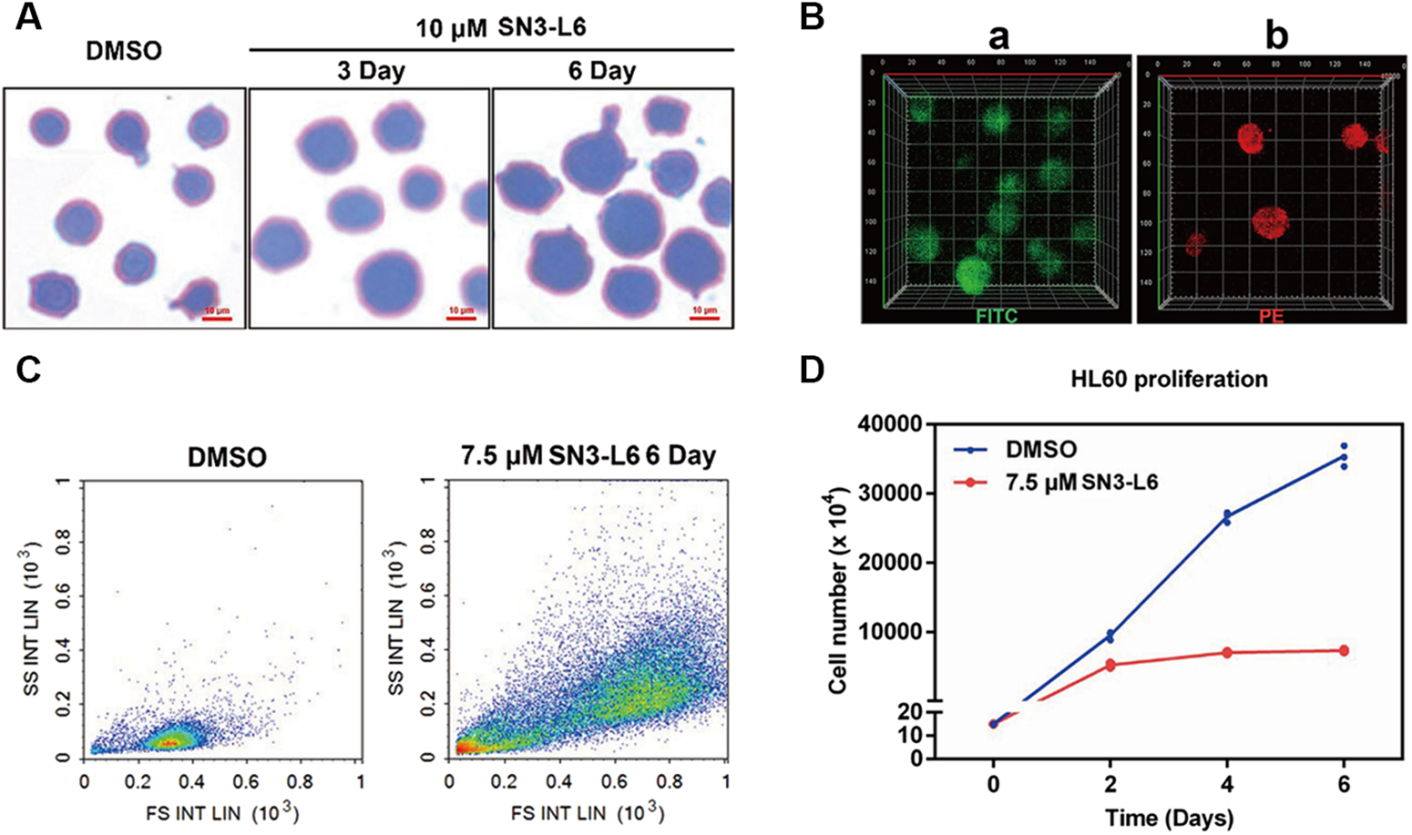
Preliminary transdifferentiation inducing activity of SN3-L6. **A** Images captured using an inverted microscope following Wright–Giemsa staining after treatment with **SN3-L6** for 3 or 6 days. **B** Images captured with a confocal microscope. Cells were co-incubated with FITC-CD41b (**a**) and PE-CD61 (**b**) following **SN3-L6** pretreatment for 9 days. Magnification ×200. **C** Sample information diagram produced from FCM assays after HL60 cells were treated with 7.5 μM **SN3-L6** or DMSO for 6 days. **D** Antiproliferation activity of **SN3-L6** against an HL60 cell line for 6 days.

An HL60 cell line, derived from an AML patient, in a suspension culture proliferates mainly (>90%) as promyelocytes. It can be induced to differentiate into functionally and morphologically mature granulocytes by compounds such as butyrate, dimethyl sulfoxide (DMSO) or hexamethylene bisacetamide^17,18^. HL60 cells were selected for our study and an interesting phenomenon was observed, that cells treated with **SN3-L6** grew larger and brighter 2 d later than did cells treated only with DMSO. A Wright–Giemsa stain assay confirmed that the cells treated with **SN3-L6** had larger nuclei (Fig. 2A). These results suggest hypothetically, that megakaryocytes may have formed after the **SN3-L6** co-incubation and flow cytometry (FCM) and immunostaining were employed to verify this hypothesis. As displayed in Fig. 2C, FCM analysis indicated that cells treated with **SN3-L6** were more complex (higher on the *Y*-axis) and larger (farther right on the *X*-axis) than untreated cells. When FITC-CD41b and PE-CD61 antibodies (the specific antigen expressed in megakaryocyte cytomembrane) were co-incubated with cells after treatment with **SN3-L6** for 9 day, labeled cells were observed fluorescently, as shown in Fig. 2B. The results indicated that the large cells produced were immunologically normal megakaryocytes. Because this is an unreported differentiation pathway, systematic studies of the transdifferentiation effect of **SN3-L6** were conducted.

### SN3-L6 inhibits proliferation of HL60 cells

Because megakaryocytes are produced upon the proliferation of HL60, we wondered whether **SN3-L6** could inhibit HL60 proliferation. HL60 cells were treated with **SN3-L6** (7.5 μM) and DMSO (<0.1%) for 6 d. It was observed that **SN3-L6** prohibited the proliferation of HL60 cells, as indicated in Fig. 2D. Cells in the group treated with **SN3-L6** had essentially ceased proliferation after 2 d, whereas those in the control group exhibited vigorous propagation. This result demonstrated that the malignant proliferation of HL60 cells was blocked by the intervention of **SN3-L6**, indicating that the cells were well controlled by **SN3-L6**.

### SN3-L6 did not result in apoptosis of megakaryocytes and granules

**Fig. 3:**
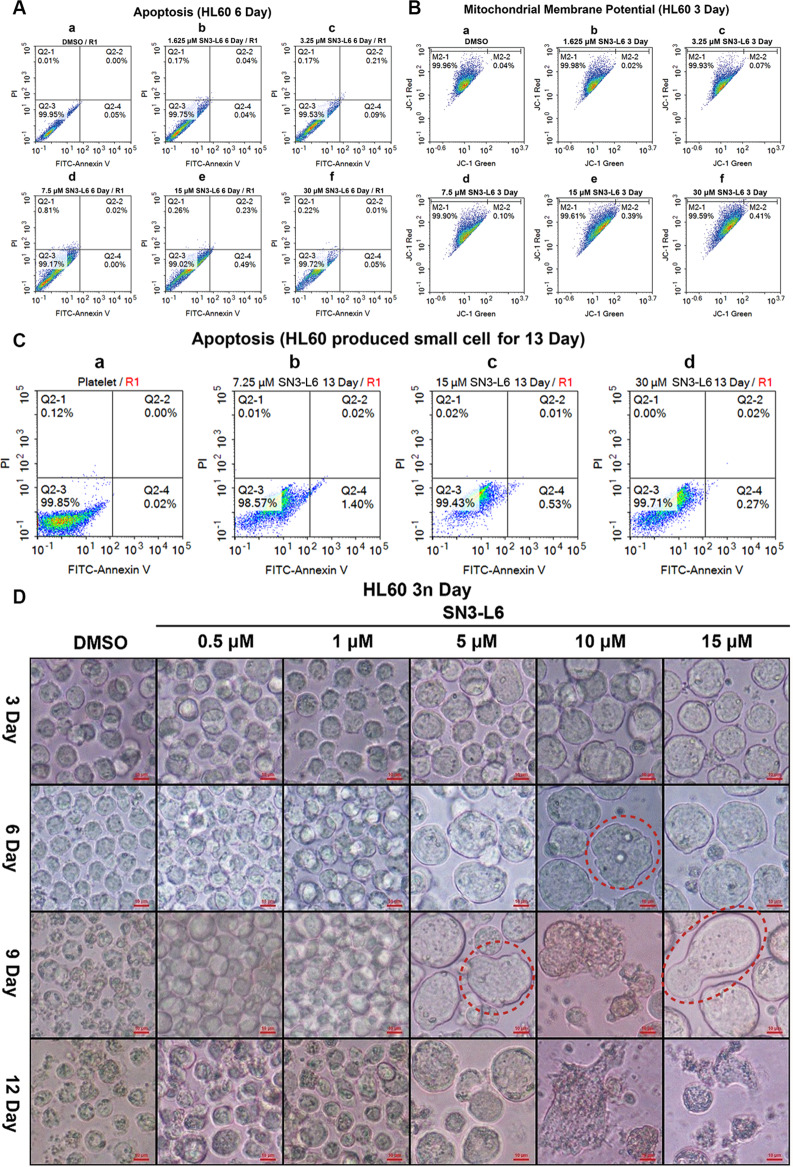
Apoptosis-related detection and cell imaging. **A** Annexin V and PI double staining assays. Cells treated with **SN3-L6** (1.625, 3.25, 7.5, 15, and 30 μM) for 6 days. **B** Mitochondrial membrane potential of HL60 cells treated with different concentrations of **SN3-L6** (1.625, 3.25, 7.5, 15, and 30 μM) for 3 day. **C** Annexin V and PI double staining assays. Granules collected the following 13 days of treatment at different concentrations of **SN3-L6** (7.5, 15, and 30 μM). **D** Images of cells treated with different concentrations of **SN3-L6** (0.5, 1, 5 μM, 10, and 15 μM) for different durations (3, 6, 9, and 12 day).

Annexin V and Propidium iodide (PI) double staining was applied to detect cell apoptosis. Figure 3A shows that the cells treated with **SN3-L6** remained alive. The absence of apoptosis indicated that this compound was an inducer with no cytotoxicity toward HL60 cells. The mitochondrial membrane potential of these cells was measured and was found to be comparable to that of the DMSO control group (Fig. 3B), which confirmed that **SN3-L6** failed to induce apoptosis in HL60 cells. The produced granules also displayed no obvious apoptosis, as seen in Fig. 3C. Our results demonstrated that the cells induced by **SN3-L6** were active and could be successfully induced to transdifferentiate.

### Cell morphologic changes after prolonged SN3-L6 treatment

**Fig. 4:**
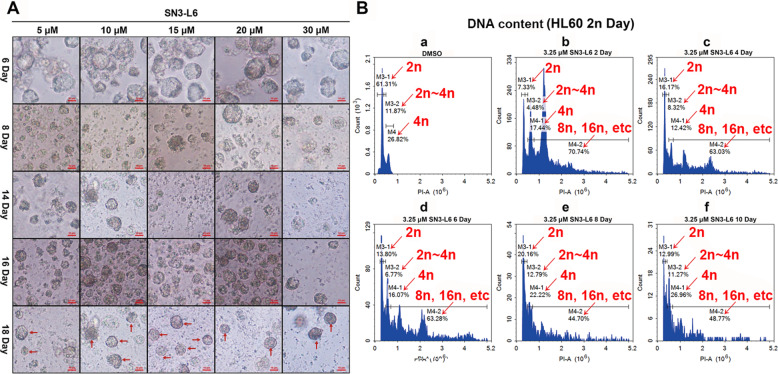
Cell imaging and cell cycle analysis. **A** Images of cells treated with different concentrations of **SN3-L6** (5, 10, 15, 20, and 30 μM) for different durations (6, 8, 14, 16, and 18 days). **B** Cell cycle analysis of HL60 cells following **SN3-L6** treatment at a concentration of 3.25 μM at day 2, day 4, day 6, day 8, and day 10.

Because cells became larger and brighter after 2 d of incubation with **SN3-L6**, we investigated the morphological changes induced by prolonged exposure to **SN3-L6**. Different concentrations (0.5, 1, 5, 10, and 15 μM) were added to cell culture flasks with a cell density <5 × 10^5^ and images were captured periodically with an inverted optical microscope. As displayed in Fig. 3D, low concentrations (0.5 and 1 μM) of **SN3-L6** were ineffective even after 12 d, whereas with higher concentrations (5, 10, and 15 μM) of **SN3-L6**, obvious morphological changes could be observed at day 3. The cells exhibited time- and concentration-dependent increases in size and brightness. On day 3, cells in the groups treated with 10 and 15 μM **SN3-L6** presented slight morphological irregularity, with ovalform, butterflyform, lageniform shapes, indicated in red dotted circles in Fig. 3D, and this phenomenon was increased considerably by day 9–15. Ameboid movement was observed on day 6, and video footage was recorded that vividly captures this strange morphologic change on day 7 at a concentration of 15 μM **SN3-L6** (Video S1 in Supplementary Information - SI). As indicated in Video S1 and Fig. 4A, the intracellular component, consisting of many granules flowed from one small chamber to another and finally, the granules were liberated into the culture medium. As shown in Fig. 4A, nearly all of the large cells had disappeared by day 14 later, leaving innumerable small cells and bare nuclei (as red arrows in Fig. 4A) distributed in the medium. This strange phenomenon is somewhat similar to the formation of platelets from a hematopoietic stem cell. The difference is that our induced differentiation course started with HL60 cells, whereas platelet formation in bone marrow starts with hematopoietic stem cells.

### SN3-L6 induces polyploidization of HL60 cells with increasing DNA content

Megakaryocytes undergo endomitotic DNA synthesis, in which DNA is produced without mitosis or cytokinesis during megakaryocyte formation^19^. Since mega-karyocytes were observed and captured, we analyzed the DNA content of cells treated with **SN3-L6**, using PI staining and FCM. As expected, increases in DNA content (M4-2 in Fig. 4B) were observed following treatment with 3.25 μM **SN3-L6** at days 2, 4, 6, 8, and 10. Cell cycle analysis revealed that the DNA content of the treated cells clearly increased and that **SN3-L6** induced HL60 cells to undergo polyploidization and endomitosis as megakaryocytes in the marrow.

### SN3-L6 elevates CD41b and CD61 expression of HL60 cells

**Fig. 5:**
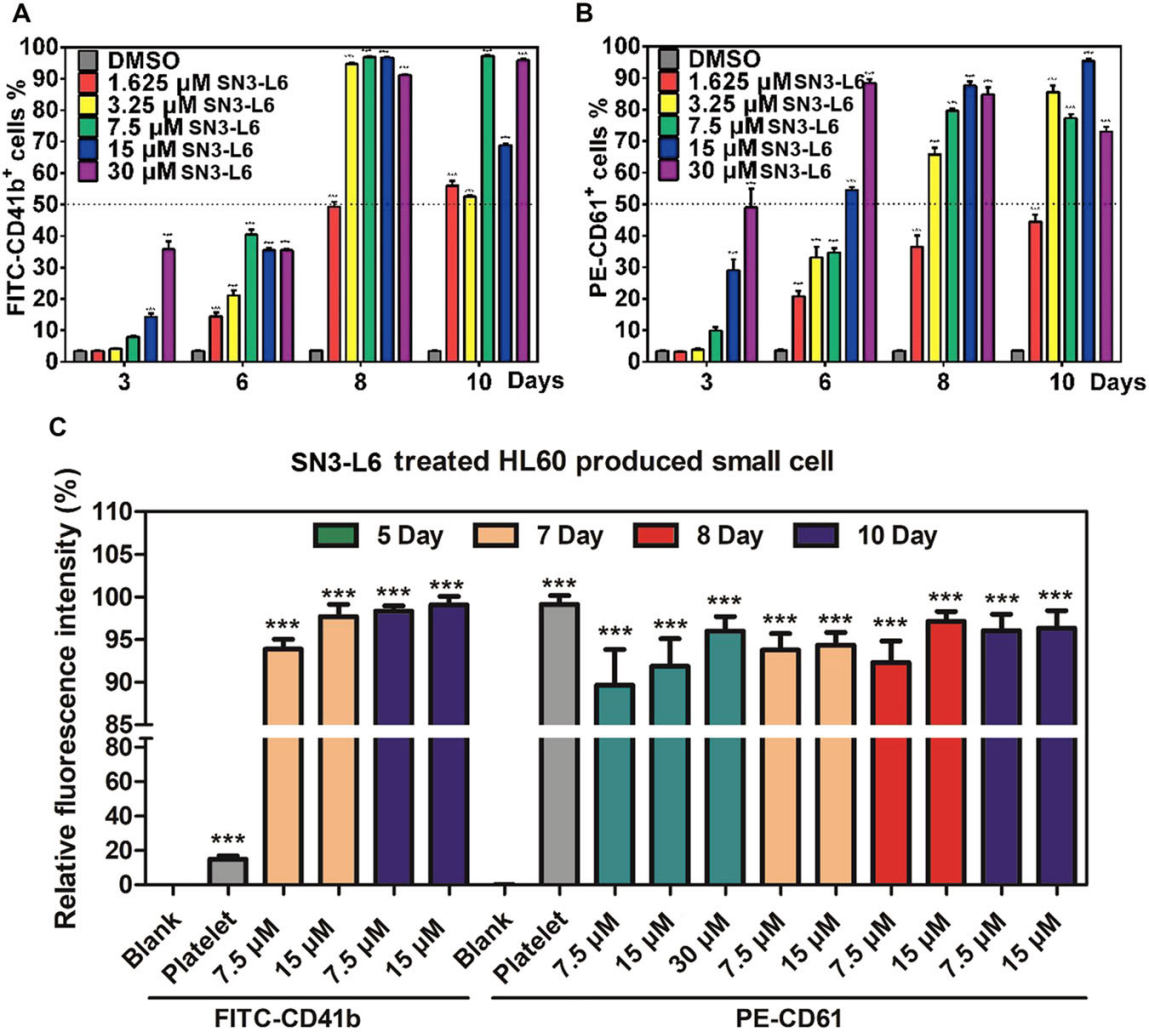
SN3-L6 exhibits transdifferentiation inducing activities with high CD41b and CD61 expression in HL60 cells. Relative CD41b and CD61 expression detection by FCM assays. Cells were collected after **SN3-L6** treatment at different concentrations (1.625, 3.25, 7.5, 15, and 30 μM) for different durations (3, 6, 8, and 10 days) and then coincubated with FITC-CD41b (**A**) or PE-CD61 (**B**) for relative fluorescence intensity detection by FCM. **C** FITC-CD41b and PE-CD61 were coincubated with granules collected from the **SN3-L6**-treated HL60 cell samples (***p < 0.001 compared with the DMSO or control group).

We deduced that micromolar levels of **SN3-L6** induce HL60 cells to differentiate into megakaryocyte cells and finally to produce granules. Because antigens such as CD41b and CD61 are expressed in megakaryocytes, we performed a quantitative analysis of CD41b and CD61 expression in cells treated with **SN3-L6**. As can be observed in Fig. 5, after co-incubation with **SN3-L6**, both CD41b (Fig. 5A) and CD61 (Fig. 5B) expression were elevated by as much as 90% in the compound-treated group compared with 3% in the DMSO-treated group. The groups treated with high concentrations of **SN3-L6** displayed significant cluster of differentiation after a short time, whereas the groups treated with low concentrations of **SN3-L6** treatment required a longer time. In addition, we collected granules after 5–10 d of treatment and measured their CD molecular expression with platelets from donated blood as a control. We found that the **SN3-L6**–induced granules had CD41b and CD61 expression as high as that of platelets (Fig. 5C). These results indicated that the megakaryocytes and granules or named platelets derived from HL60 cells treated with **SN3-L6** had normal immunity. At this point, we were able to draw the conclusion that HL60 cells were induced to transdifferentiate into megakaryocytes and platelets by **SN3-L6**. To the best of our knowledge, this is a novel transdifferentiation pathway from HL60 cells and is entirely different from the reported differentiation pathways of HL60 cells shown in Fig. 1.

### SN3-L6 does not induce the differentiation of mature granulocytes into megakaryocytes

**Fig. 6:**
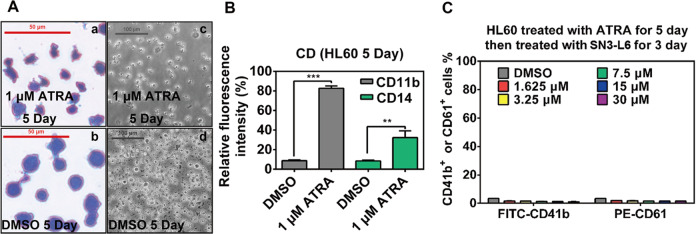
Mature granulocyte model construction and CD41b and CD61 detection. **A** Images from an inverted microscope after DMSO or 1 μM **ATRA** treatment (**c** and **d**) and Wright–Giemsa staining assay (**a** and **b**). **B** Relative CD11b and CD14 expression after DMSO or 1 μM **ATRA** treatment. **C** Relative CD41b and CD61 expression after DMSO or 1 μM **ATRA** pretreatment with subsequent **SN3-L6** treatment (1.625, 3.25, 7.5, 15, and 30 μM) for 3 days (**p < 0.01, ***p < 0.001 compared with the DMSO group).

For an inducing agent, selectivity is crucial. ATRA-treated HL60 cells were used to investigate if **SN3-L6** affects mature granulocytes. As shown in Figs. 6A, 1 μM of **ATRA**-treated HL60 cells exhibited reduced cell size but an enlarged cytoplasm, resulting in a low nucleus/cytoplasm ratio. The FCM assay also revealed that ATRA-treated cells displayed high expression of CD11b and CD14 (Fig. 6B), two specific biomarkers of mature granulocytes. When **SN3-L6** was added to HL60 cells pretreated with 1 μM **ATRA** for 3 d, no obvious CD41b or CD61 expression was detected (Fig. 6C). This implies that **SN3-L6** does not induce differentiation of mature granulocytes into megakaryocytes, and therefore **SN3-L6** displays selectivity between immature and mature cells.

### SN3-L6 induces K562 cells to differentiate into megakaryocytes but fails to produce platelets

**Fig. 7:**
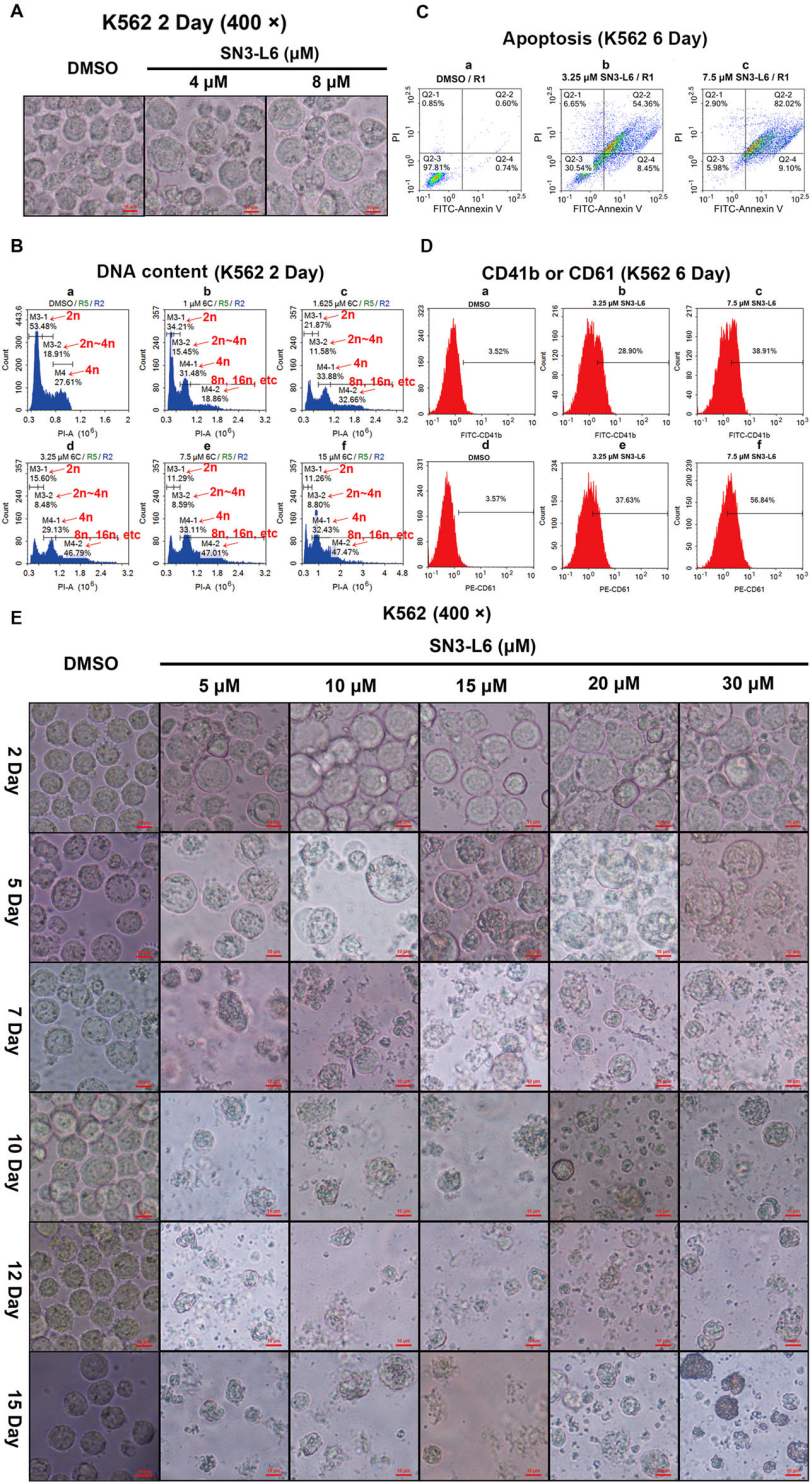
SN3-L6 induces K562 cells transdifferentiate into megakaryocyte without platelets production. **A** Images of K562 cells treated with different concentrations of **SN3-L6** (4 and 8 μM) for 2 days. **B** DNA content detection for K562 cells treated with different concentrations of **SN3-L6** (1, 1.625, 3.25, 7.5, and 15 μM) for 2 days. **C** Apoptosis analysis for K562 cells treated with DMSO, 3.25 and 7.5 μM of **SN3-L6** for 6 days. **D** CD41b and CD61 expression detection of K562 cells treated with DMSO, 3.25 and 7.5 μM **SN3-L6** for 6 days. **E** Images of K562 cells treated with different concentrations of **SN3-L6** (5, 10, 15, 20, and 30 μM) at day 2, day 5, day 7, and day 10.

Since we have observed the selectivity of **SN3-L6**, we sought to learn whether **SN3-L6** can induce different kinds of leukemia cells to differentiate into platelets. K562, a typical type of CML^20^ was selected to investigate this possibility. On day 2, K562 cells treated with **SN3-L6** displayed deep morphological changes, growing larger and brighter (Fig. 7A). Cell cycle analysis revealed that high DNA content (M4-2 in Fig. 7B) became obvious after co-incubation with **SN3-L6** and CD41b and CD61 were also augmented in the K562 cells treated with **SN3-L6** (Fig. 7D). The disappearance of megakaryocytes tended to diminish at day 5 with none of the ameboid movement and platelet production that had been observed in **SN3-L6** treated HL60 cells (Fig. 7E) was seen. K562 cells became apoptotic when treated for 6 days with 3.25 μM and 7.5 μM of **SN3-L6** (Fig. 7C). We observed that **SN3-L6** could induce K562 to transdifferentiate into megakaryocytes instead of sequential platelet formation, with subsequent shrinkage and apoptosis. It has been reported that K562 cells can be induced to transdifferentiate to megakaryocytes^14–16^. We found that **SN3-L6** also induces K562 cells to transdifferentiate into megakaryocytes as indicated in Fig. 1. It is interesting that different kinds of leukemia cells and mature blood cells respond so differently to **SN3-L6**, indicating the precise targets in HL60 cells of this compound.

### Preliminary exploration of the mechanism

**Fig. 8:**
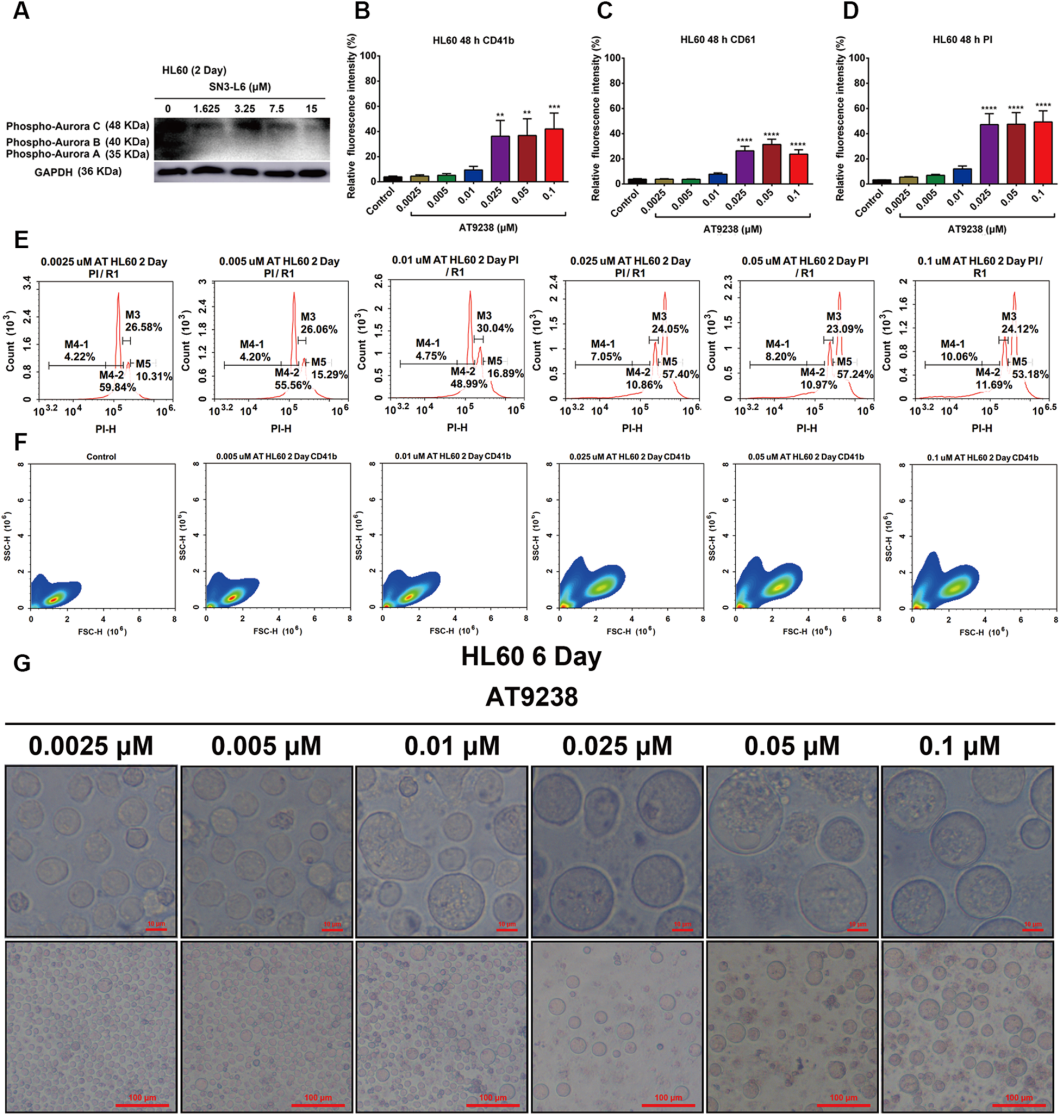
Preliminary exploration of the mechanism. **A** Western blot assay of HL60 cells treated with different concentrations of **SN3-L6** (1.625, 3.25, 7.5, 15, and 30 μM) for 2 days. **B** CD41b detection of HL60 cells after treatment with different concentrations of **AT9238** (0.0025, 0.005, 0.01, 0.025, 0.05, and 0.1 μM). **C** CD61 detection of HL60 cells after treatment with different concentrations of **AT9238** (0.0025, 0.005, 0.01, 0.025, 0.05, and 0.1 μM). **D** PI staining of HL60 cells after treatment with different concentrations of **AT9238** (0.0025, 0.005, 0.01, 0.025, and 0.1 μM). **E** Cell cycle analysis of HL60 cells following with different concentrations of **AT9238** (0.0025, 0.005, 0.01, 0.025, 0.05, and 0.1 μM) for 2 days. **F** Sample information diagram produced from FCM assays after HL60 cells were treated with different concentrations of **AT9238** (0.005, 0.01, 0.025, 0.05, and 0.1 μM) for 2 days. **G** Images of HL60 cells treated with different concentrations of **AT9238** (0.0025, 0.005, 0.01, 0.025, 0.05, and 0.1 μM) for 6 day. (**p < 0.01, ***p < 0.001, ****p < 0.0001 compared with the control group).

Since earlier reports have revealed that inhibition of aurora kinases results in endomitosis^21^, we tested the aurora kinase expression in HL60 cells after exposure to **SN3-L6**. The results indicated that **SN3-L6** inhibited phospho-aurora kinase A and B expression in HL60 cells (Fig. 8A), which suggests that **SN3-L6** may induce differentiation through this pathway. The aurora kinase A and B dual inhibitor **AT9283**^22^ were selected to determine whether it exhibited the same effects as **SN3-L6**. FCM analysis (Fig. 8F) indicated that **AT9283**–treated cells are more complex (higher on the Y-axis) and larger (farther right on the *X*-axis). Expression of obvious megakaryocytic biomarkers CD41b (Fig. 8B), CD61 (Fig. 8C), and DNA content of **AT9283**–treated cells (Fig. 8E) were detected. However, although **AT9283** induced HL60 cells to transdifferentiate into megakaryocytes (Fig. 8G), these megakaryocytes displayed strong PI staining at day 2 (Fig. 8D), indicating that **AT9238** induced HL60 cells were necrotic or mid and late apoptotic, similar to megakaryocytes derived from human erythroleukemia cells^23^. These results suggested that the aurora kinase pathway is likely to be one of many possible targets of **SN3-L6**. Multitargeting by **SN3-L6** may be a key factor underlying its special transdifferentiation activity.

## Discussion

The current study is the first to demonstrate that AML HL60 cells can be chemically induced to transdifferentiate into morphologically and immunologically normal megakaryocytes and platelets. Specifically, the securinine derivative **SN3-L6** can induce HL60 cells to transdifferentiate into platelets. Our data indicated that after 2 d treatment with **SN3-L6**, cells are well controlled, with a low proliferation rate in a 7.5 μM **SN3-L6** rich medium, and are gradually induced to transdifferentiate into megakaryocytes. More importantly, **SN3-L6** failed to cause apoptosis in the megakaryocytes that were differentiated from HL60 cells, and for approximately one week, it effectively promoted platelet production. Cytoplasm, DNA content and morphological maturation features were markedly increased in the induced cells. Moreover, CD41b and CD61, markers of the megakaryocyte phenotype, were highly expressed in cells treated with **SN3-L6**, indicating that the megakaryocytes and platelets were immunologically normal. Finally, **SN3-L6** failed to induce mature granulocytes to transdifferentiate into megakaryocytes, let alone platelets. **SN3-L6** can drive CML K562 cells to transdifferentiate into megakaryocytes without the formation and release of platelets.

In summary, the results of this study are significant in blood cell death and leukemia treatment. These results constitute the first discovery of megakaryocytic transdifferentiation of HL60 cells to platelets. Megakaryocytes and erythrocytes both stem from megakaryocyte-erythroid progenitors^24,25^ and K562 cells displaying a differentiation block at the immature erythrocyte stage are easily induced into megakaryocytes biologically. Our newly discovered transdifferentiation pathway implies that HL60 cells and megakaryocytes or platelets are biologically correlated. Cells originating between them may be closer than previously thought, and this should be studied in the future to reveal the extent of the similarity. Secondly, as has been reported, the induction of polyploidization and differentiation of acute myeloid megakaryocyte leukemia may be an effective strategy for the treatment of acute myeloid megakaryocyte leukemia^26^. Our results imply that the induction of polyploidization may be an effective anti-leukemia strategy, as platelets and megakaryocytes will die ultimately. Our future research will involve the investigation of the biological function of platelets and in vivo anti-leukemia activity using **SN3-L6**. We also hope in the near future to reveal the molecular mechanism underlying this discovery.

## Materials and methods

### Cell culture

HL60 cells and K562 cells were cultured in RPMI 1640 (Gibco) medium containing 10% fetal bovine serum (Gibco), 100 IU/mL penicillin, and 0.1 mg/mL streptomycin (Gibco) in a humidified circumstance with 5% CO_2_ at 37 ^o^C. When cell density is >5 × 10^5^, samples should be diluted to promote a healthy cellular status. Cells in the exponential growth phase are the best fit for all the in vitro experiments.

### Wright–Giemsa stain assay

Cells treated with **SN3-L6** (10 μM) or DMSO for 3 or 6 days were collected and washed three times with phosphate buffer saline (PBS). A cell suspension was coated on a glass slide. Then Wright–Giemsa solution A (Beso Biotechnology Company, Zhuhai, China) was added for 3 min and solution B (Beso Biotechnology Company, Zhuhai, China) was added for another 2 min ensuring their blending. Samples were washed gently with water before being captured on an inverted microscope. This was repeated three times for each sample.

### Anti-proliferation activity of SN3-L6 towards HL60 cells

1.5 × 10^5^ cells were seeded in a cell culture flask and treated with **SN3-L6** (7.5 μM) or DMSO for 6 days. Cell numbers were counted every second day. When the density exceeds 5 × 10^5^, samples should be diluted. Repeat three times for each sample and subject data to GraphPad Prism 6 to get the final results.

### FCM assays

Cells were collected and washed using PBS three times after compound or DMSO treatment on different days according to the requirements. For cell cycle detection, cells were fixed by 70% EtOH overnight at 4 ^o^C. PI solution (200 μL) containing RNAase (Thermo) was added, the mixture was incubated for 15 min, then samples were filtered (40–50 μm nylon net) before detection. FITC Annexin V-PI Apoptosis Detection Kit I (BD Biosciences, 556547) was used for apoptosis detection. The procedures were as follows: 50 μL binding buffer, 2.5 μL PI, and 2.5 μL Annexin V were added to resuspended samples for 15 min. Then 150 μL binding buffer were added before centrifugation. Finally, the cells were resuspended in 200 μL binding buffer for FCM analysis. For CD11b (BD Biosciences, 555388), CD14 (BD Biosciences, 555397), CD41b (BD Biosciences, 555469), and CD61 (BD Biosciences, 555754) detection, antibodies were added to cells prior to incubation for 30 min, then they were washed by PBS three times before FCM detection. A mitochondrial membrane potential detection kit (BD Biosciences, USA) was selected for mitochondrial membrane potential detection and used according to the manufacturer’s instructions. FCM assays were conducted on FCM (ACEABIO | NovoCyte or BD FACSCanto, USA). Repeat three times for each sample.

### Western blot assay

The western blot experimental procedure is as previously described^11^. The antibodies used in this case are Phospho-Aurora (Beyotime Biotechnology, AA923-1). Repeat three times for each sample.

### Video and images capturing assay

The video was captured with a live cell workstation (Leica, Germany). Laser confocal pictures were obtained by laser scanning confocal microscope (Zeiss, Germany). Cellular morphology images are taken by ordinary optical microscope (Leica, Germany). Repeat three times for each sample.

### Basic information for compounds

**SN3-L6** studied in this research was reported previously^12^ and **AT9238** was purchased from TargetMol Company (catalog number T3068).

## Supplementary information

Morphologic change of HL60 cells

## References

[1] Breitman TR, Collins SJ, Keene BR (1981). Terminal differentiation of human promyelocytic leukemic cells in primary culture in response to retinoic acid. Blood.

[2] Huang ME (1988). Use of all-trans retinoic acid in the treatment of acute promyelocytic leukemia. Blood.

[3] Huang ME (1987). All-trans retinoic acid with or without low dose cytosine arabinoside in acute promyelocytic leukemia. Report of 6 cases. Chin. Med. J..

[4] Shen ZX (2004). All-trans retinoic acid/As_2_O_3_ combination yields a high quality remission and survival in newly diagnosed acute promyelocytic leukemia. Proc. Natl Acad. Sci. USA.

[5] Breitman TR, Selonic SE, Collins SJ (1980). Induction of differentiation of the human promyelocytic leukemia cell line (HL-60) by retinoic acid. Proc. Natl Acad. Sci. USA.

[6] Ferrara F, Schiffer CA (2013). Acute myeloid leukaemia in adults. Lancet.

[7] Nowak D, Stewart D, Koeffler HP (2009). Differentiation therapy of leukemia: 3 Decades of development. Blood.

[8] Wang XN (2014). ERK 5/MAPK pathway has a major role in 1 alpha,25-(OH)_2_ vitamin D-3-induced terminal differentiation of myeloid leukemia cells. J. Steroid Biochem. Mol. Biol..

[9] Wang X, Wang TT, White JH, Studzinski GP (2006). Induction of kinase suppressor of RAS-1(KSR-1) gene by 1, alpha25-dihydroxyvitamin D3 in human leukemia HL60 cells through a vitamin D response element in the 5’-flanking region. Oncogene.

[10] Dipak Das GP (2000). Arnold Stern MAPK-dependent expression of p21^WAF^ and p27^kip1^ in PMA-induced differentiation of HL60 cells. FEBS Lett..

[11] Hou W (2019). Novel virosecurinine bivalent mimetics as potent reversal agents against P-glycoprotein-mediated multidrug resistance. Eur. J. Med. Chem..

[12] Tang G (2016). Design and synthesis of dimeric securinine analogues with neuritogenic activities. ACS Chem. Neurosci..

[13] Liao YM (2018). A bivalent securinine compound SN3-L6 induces neuronal differentiation via translational upregulation of neurogenic transcription factors. Front. Pharmacol..

[14] Sutherland JA, Turner AR, Mannoni P, McGann LE, Turc JM (1986). Differentiation of K562 leukemia cells along erythroid, macrophage, and megakaryocyte lineages. J. Biol. Response Mod..

[15] Shelly C, Petruzzelli L, Herrera R (2000). K562 cells resistant to phorbol 12-myristate 13-acetate-induced growth arrest: dissociation of mitogen-activated protein kinase activation and Egr-1 expression from megakaryocyte differentiation. Cell Growth Differ..

[16] Tetteroo PA, Massaro F, Mulder A, Schreuder-Van GR, Ae VDB (1984). Megakaryoblastic differentiation of proerythroblastic K562 cell-line cells. Leuk. Res..

[17] Breitman TR, Selonick SE, Collins SJ (1980). Induction of differentiation of the human promyelocytic leukemia cell line (HL-60) by retinoic acid. Proc. Natl Acad. Sci. USA.

[18] Jiang G, Albihn A, Tang T, Tian Z, Henriksson M (2008). Role of Myc in differentiation and apoptosis in HL60 cells after exposure to arsenic trioxide or all-trans retinoic acid. Leuk. Res..

[19] Mazzi S, Lordier L, Debili N, Raslova H, Vainchenker W (2018). Megakaryocyte and polyploidization. Exp. Hematol..

[20] Lozzio CB, Lozzio BB (1975). Human chronic myelogenous leukemia cell-line with positive Philadelphia chromosome. Blood.

[21] Moore AS, Blagg J, Linardopoulos S, Pearson ADJ (2010). Aurora kinase inhibitors: novel small molecules with promising activity in acute myeloid and Philadelphia-positive leukemias. Leukemia.

[22] Vormoor B (2017). A phase I/II trial of AT9283, a selective inhibitor of aurora kinase in children with relapsed or refractory acute leukemia: challenges to run early phase clinical trials for children with leukemia. Pediatr. Blood Cancer.

[23] Ghezali L, Liagre B, Limami Y, Beneytout JL, Leger DY (2014). Sonic Hedgehog activation is implicated in diosgenin-induced megakaryocytic differentiation of human erythroleukemia cells. PLoS ONE.

[24] Psaila B (2016). Single-cell profiling of human megakaryocyte-erythroid progenitors identifies distinct megakaryocyte and erythroid differentiation pathways. Genome Biol..

[25] Shinohara A (2014). Intracellular reactive oxygen species mark and influence the megakaryocyte-erythrocyte progenitor fate of common myeloid progenitors. Stem Cells.

[26] Wen Q (2012). Identification of regulators of polyploidization presents therapeutic targets for treatment of AMKL. Cell.

